# Supplementary material for: Content-rich biological network constructed by mining PubMed abstracts
Source: BMC Bioinformatics. 2004 Oct 8;5:147. doi: 10.1186/1471-2105-5-147 (PMC528731; doi:10.1186/1471-2105-5-147)
Supplement: Additional File 2 — The original results of the above study (non-essential files are deleted to keep the file size under the limit set by BMC bioinformatics). [file 1471-2105-5-147-S2.bz2 › chilibotAdditionalFile2/dip05/658ID10525412E11049/html/NIFD_NIFK.html]

 


 **NIFD** and **NIFK** 
  
Found 195 abstracts in PubMed, retrieved 05.  
 

 What does Google say? 
 PDF only 
| .edu only 

---

**Interactive relationship** (e.g. stimulation, inhibition, etc)

**Non-interactive relationship** (e.g. studied together, co-existance, homology, etc.)

- As such this represents a new fold capable of binding FeMo co, where the only previous example was that seen in dinitrogenase  [ **NIFK** ]   [ **NIFD** ] .  Ref: 12754195 J Biol Chem< MedlineTA>J Biol Chem,
- VnfY is required for full activity of the vanadium containing dinitrogenase  [ **NIFK** ]   [ **NIFD** ]  in Azotobacter vinelandii.  Ref: 12644512 J Bacteriol, 2003
- A mutant strain having an insertion mutation in vnfY has 10 fold less vnf dinitrogenase  [ **NIFK** ]   [ **NIFD** ]  activity and exhibits a greatly diminished level of 49 V label incorporation into the V dependent dinitrogenase  [ **NIFK** ]   [ **NIFD** ]  when compared to the wild type.  Ref: 12644512 J Bacteriol, 2003
- These results indicate that VnfY has a role in the maturation of the V dependent dinitrogenase  [ **NIFK** ]   [ **NIFD** ] , with a specific role in the formation of the V containing cofactor and or its insertion into apodinitrogenase.  Ref: 12644512 J Bacteriol, 2003
- Although restriction analysis of the  **nifD**   **nifK**  intergenic DNA fragment confirmed the unique nature of Rhizobium sp.  Ref: 12802480 Arch Microbiol< MedlineTA>Arch Microbiol, 2003
- The MoFe protein component of the complex metalloenzyme nitrogenase is an alpha2beta2 tetramer encoded by the  **nifD**  and the  **nifK**  genes.  Ref: 12468552 J Biol Chem, 2003
- Significant amounts of both dinitrogenase  [ **NIFK** ]   [ **NIFD** ]  and dinitrogenase  [ **NIFK** ]   [ **NIFD** ]  reductase were only formed when the growth medium was supplemented with molybdate 1 microM.  Ref: 12667075 Biochemistry, 2003
